# Indirect reference intervals for TSH in a sample of lebanese pregnant women

**DOI:** 10.1016/j.plabm.2025.e00460

**Published:** 2025-03-08

**Authors:** Dollen Eid, Nizar El Bcherawi, Georges Abi Tayeh, Nada El Ghorayeb, Marie-Hélène Gannagé-Yared

**Affiliations:** a Department of Endocrinology, Saint Joseph University, Faculty of Medicine, Beirut, Lebanon; b Department of Gynecology and Obstetrics, Saint Joseph University, Faculty of Medicine, Beirut, Lebanon

**Keywords:** Thyroid-stimulating hormone (TSH), Pregnancy, Indirect reference intervals (RIs), Maternal thyroid hormones, Thyroid disorders

## Abstract

**Background:**

Thyroid dysfunction in pregnant women can lead to fetal complications. Thyroid-stimulating hormone (TSH) is the key hormone for diagnosis of thyroid dysfunction. No previous study has established reference intervals (RI) for TSH in Lebanese pregnant women. The objective of this study is to define the TSH RIs for each trimester of pregnancy in healthy Lebanese pregnant women using an indirect method.

**Materials and methods:**

This retrospective study included 287 pregnancies selected from the records of an obstetric clinic at Hôtel-Dieu de France University Hospital from January 2021 to May 2023. A control group of 103 non-pregnant women was also included in the study. The collected TSH values were stratified by trimester (first and second) of pregnancy and postpartum. After applying the exclusion criteria, a total of 458 TSH values were included in the analysis.

**Results:**

The respective medians and RIs for TSH during the first, second pregnancy trimesters and postpartum are 1.57 (0.43–3.20 mIU/L), 1.84 (0.56–4.41 mIU/L), and 1.38 (0.30–3.60 mIU/L), while for the control group it is 1.66 (0.64–4.24 mIU/L). There is a significant correlation between TSH values in the first trimester and those in the second trimester and postpartum (p ≤ 0.001 and p = 0.002 respectively). No significant correlation was observed between age and TSH levels in the first and second trimesters and as well as in postpartum.

**Conclusion:**

Our RIs are close to the revised American Thyroid Association (ATA) recommendations. Further research is needed to understand the mechanisms and clinical impact of these differences.

## 1 Introduction

Thyroid hormones (TH) are crucial for fetal development, particularly during the first trimester of pregnancy. Although the fetal thyroid gland is present and functional at 10–12 weeks of gestation, it does not fully mature until 18–20 weeks of gestation [1]. Consequently, the fetus depends on maternal hormones delivered through the placenta early in gestation. These hormones play a critical role in placental development and in the growth and maturation of various fetal tissues, especially the brain and the skeleton. Thyroid dysfunctions are more prevalent in pregnant women compared to the general population and can negatively impact fetal development, potentially leading to miscarriages, preterm births, preeclampsia, and lower intellectual quotient (IQ) in children [2]. Hypothyroidism occurs in approximatively 4 % of pregnancies (0.5 % clinical hypothyroidism and 3.5 % subclinical hypothyroidism), while hyperthyroidism in 2.4 % of pregnancies (0.6 % clinical hyperthyroidism and 1.8 % subclinical hyperthyroidism) [3]. Surprisingly, in Lebanon, a previous study estimated the prevalence of hypothyroidism in pregnant women to 17 % [4].

Establishing trimester-specific reference intervals (RIs) for thyroid-stimulating hormone (TSH) is essential and aligns with international recommendations for monitoring of pregnant women [2]. The American Thyroid Association (ATA) previously defined TSH reference values as 0.1–2.5, 0.2–3.0, and 0.3–3 mIU/L for the 1st, 2nd and 3rd trimester of pregnancy. In 2017 the ATA recommendations were revised and normal TSH values were redefined in pregnancy as being less than 4 mIU/L over the three trimesters [5]. Several reasons exist behind this change: first, the ATA recognized significant variability in TSH levels across populations due to differences in iodine intake, genetic factors, and assay methodologies. Therefore, a fixed cutoff value for the upper limit was necessary to provide clearer clinical guidance in the absence of local data. In addition, large studies had shown that the risk of adverse pregnancy outcomes significantly increased when TSH levels exceeded 4.0 IU/L. A cutoff of 4.0 IU/L simplifies clinical management when local trimester-specific RI are unavailable. Specific RIs vary based on ethnicity, iodine status, gestational age, body mass index (BMI), and TSH assay [6,7]. Thyroid peroxidase antibodies (TPO-Ab) and thyroglobulin antibodies (TG-Ab) are also associated with higher TSH values during pregnancy [3]. Specific and adapted RIs enable better detection and management of pregnancy thyroid disorders, and subsequently prevention of fetal consequences through appropriate treatment. In the recent metanalysis by Osinga et al. [8], the authors suggested that relying on alternative approaches to define pregnancy RIs for TSH -such as non-pregnant RIs, fixed upper limits for TSH of 4 mIU/L, or non-pregnancy RIs with a 0.5 mIU/L subtraction from the TSH upper limit-result in considerable overdiagnosis and underdiagnosis compared with population and trimester-specific RIs.

While trimester-specific RIs have been determined in various populations, including the United Arab Emirates (UAE) [9,10], China [11], India [12], Europe [13], and the United States (US) [14] and others [15,16], no study has been conducted in the Lebanese population. The objective of this study is to define TSH RIs for the first and second trimesters of pregnancy in healthy Lebanese pregnant women using an indirect approach [17]. This method allows for the determination of RIs tailored to the specific population under study.

## 2 Materials and methods

### 2.1 Population

**Fig. 1:**
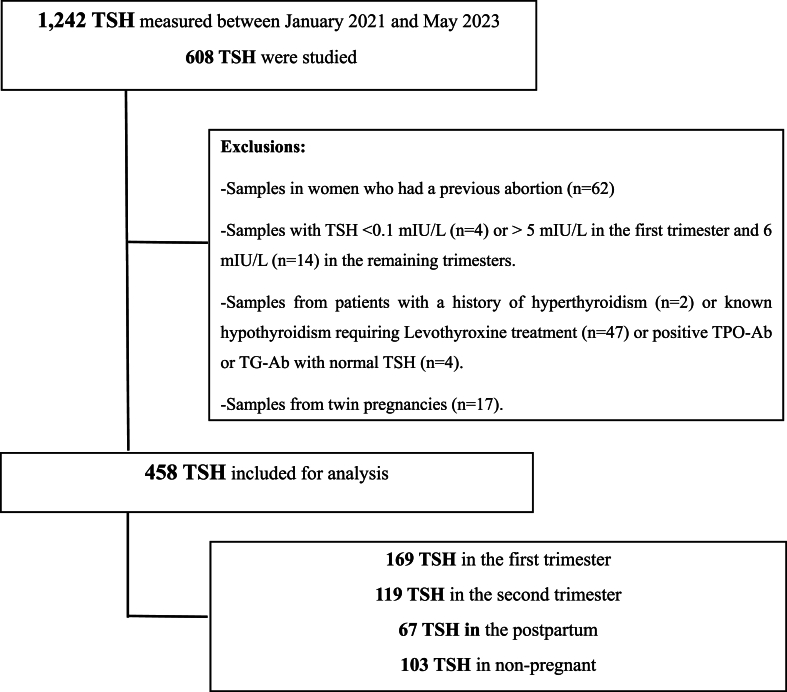
Flowchart illustrating the selection criteria for estimating RI of TSH.

The medical records of all patients (pregnant and non-pregnant) aged between 18 and 49 who visited an obstetric clinic at Hôtel-Dieu de France University Hospital from January 2021 to May 2023 were retrospectively examined. Only women who underwent a TSH measurement were included in the study. Variables such as age, number of pregnancies, parity, previous abortions, current medications, medical history (including history of thyroid disorders, and previous measurements of TPO-Ab and/or TG-Ab were recorded. Exclusion criteria included previous abortion, known thyroid disorders, previous thyroidectomy or radioiodine treatment, use of medications affecting thyroid function (levothyroxine, antithyroid drugs), known positive thyroid autoimmunity (TPO-Ab strictly greater than 34 IU/mL and/or TG-Ab strictly greater than 100 IU/mL), TSH values > 5 mIU/L or < 0.1 mIU/L in the first trimester and >6 mIU/L or < 0.1 mIU/L in the second trimester. These cutoffs were chosen to exclude aberrant values based on the boxplots. 14 values were removed from the analysis because they were mild outliners (between 1.5 and 3 interquartile range) or extreme outliners (beyond 3 interquartile range). A total of 477 records were analyzed, from which 150 were excluded from the study: 62 due a previous history of abortion (10.2 %), 47 for a history of hypothyroidism (7.73 %), two for a history hyperthyroidism (0.33 %), 4 (0.66 %) for positive TPO-Ab, 17 because of twin pregnancies (2.80 %) and finally 18 aberrant values (3.74 %). A total of 327 women were finally selected for the study. Among them, 224 were pregnant and 103 non pregnant constituting the control group. Most of pregnant women had multiple TSH values recorded at different stages of pregnancy (first and second trimesters), and/or postpartum. As a result, the total count of TSH values is 458, which includes 103 values from non-pregnant women and 355 values from pregnant women across the first and second trimester of pregnancy and postpartum stage Fig. 1.

### 2.2 Ethical considerations

The study protocol was approved by the Ethics Committee of Saint Joseph University in Beirut, the university affiliated hospital being Hȏtel-Dieu de France (code Tfem/2023/24). No informed consent was needed since the data were collected from medical records.

### 2.3 TSH assay

TSH RI was established using the indirect method according to the Jones et al.’s recommendations [17]. TSH levels were measured using a Cobas e411 electrochemiluminescence analyzer (Roche Diagnostics, Mannheim, Germany) using the Elecsys cobas e 200 V2 reagent. The RI provided by the manufacturer for the TSH assay was from 0.27 to 4.2 mIU/L, the **Lower Detection Limit**: 0.005 μIU/mL and the functional sensitivity 0.014 mIU/L. TSH values were categorized based on gestational age: those between 0 and 14 weeks were classified as the first trimester, those between 14 and 28 weeks as the second trimester, and those from 28 weeks until delivery as the third trimester [18]. TSH values measured in women from the delivery date up to two months postpartum were categorized as the postpartum period. Some women had multiple TSH measurements at different stages of pregnancy (first, second, and third trimesters) and/or during the postpartum period.

### 2.4 Statistical method

The variables were entered into an Excel file. Statistical analysis was performed using the SPSS software (IBM Corp; SPSS Statistics for Windows v26.1, Armonk, NY, USA) and the GraphPad Prism 8.00 (GraphPad Prism Software, Inc. La Jolla, USA). The distribution of quantitative variables was checked using Kolmogorov–Smirnov (KS) and Shapiro–Wilk (SW) tests. Categorical (qualitative) variables were expressed as percentages and frequencies (counts), while quantitative variables with non-normal distribution were presented as median with interquartile range (quartile 1 to 3). Correlations between variables were estimated using the Spearman correlation coefficient in case of significant deviation from normality in at least one of the two variables. Kruskal–Wallis (KW) test was used for non-normally distributed variables to compare TSH values between the first 2 pregnancy trimesters and postpartum. P values < 0.05 were considered statistically significant.

## 3 Results

### 3.1 Population characteristics

The age of pregnant women ranged from 22 to 44 years, while the age of the control group ranged from 18 to 49 years. Regarding the distribution of the number of pregnancies and parity among the sample of 224 pregnant women, most of the women were on their first pregnancy (58.9 %), followed by those in their second pregnancy (35.3 %). A smaller proportion of tests were recorded for third and fourth pregnancies (5.4 % and 0.4 %, respectively). A significant proportion of women were nulliparous (54.9 %), followed by those who were primiparous (36.6 %) and those with Parity 2 (7.6 %). 0.9 %% of women had three children. 62 women had aborted at least once and were excluded from the analysis. In the overall population only 33 women have a previous TPO-Ab or TG-Ab measurements.

### 3.2 Distribution of TSH values among the studied population

Table 1 presents the numbers and frequencies (%) of TSH values, along with the age of the women who underwent these measurements during the first and second trimester of pregnancy, and in the postpartum. Data from non-pregnant women are also presented in this table. A total of 458 TSH values were recorded.

**Table 1:** Thyroid-stimulating hormone (TSH) percentiles and confidence intervals across pregnancy stages and non-pregnant group.

Pregnancy Stage	N (%)	Age Range (Mean ± SD)	TSH Percentiles (2.5, 50, 97.5)	TSH CI for Median (50th Percentile)
**First Trimester**	169 (36.9 %)	22–43 (32.64 ± 4.28)	0.43, 1.57, 3.20	(1.542, 1.788)
**Second Trimester**	119 (26.0 %)	22–44 (33.95 ± 3.80)	0.56, 1.84, 4.41	(1.774, 2.067)
**Postpartum**	67 (14.6 %)	22–44 (33.21 ± 4.06)	0.30, 1.38, 3.60	(1.187, 1.582)
**Non-pregnant**	103 (22.5 %)	18–49 (34.04 ± 6.73)	0.64, 1.66, 4.24	(1.602, 1.918)

**CIs**: Confidence intervals.

Age is expressed as mean ± standard deviation (Sd Deviation).

Respectively during the first and second trimesters, 169 (36.94 %) and 119 (26.0 %) TSH values were measured while in the postpartum it was 67 TSH values (14.6 %). The respective average age in the women in the first and second trimester groups and in the post-partum is 32.64 ± 4.28, 32.95 ± 3.8 and 33.21 ± 4.06 years. The median gestational age in the first and second trimesters is 5.7 weeks (range: 3–13.6 weeks) and 24.9 weeks (range: 16.4–27.4 weeks), respectively. Finally, 103 non-pregnant women (22.5 %) had TSH measurements with an average age of 34.04 ± 6.73 years (Table 1). There is no significant age difference between the two pregnancy trimesters, postpartum, and non-pregnant women (p = 0.2).

### 3.3 RIs for TSH by trimester, postpartum, and in non-pregnant patients

**Fig. 2:**
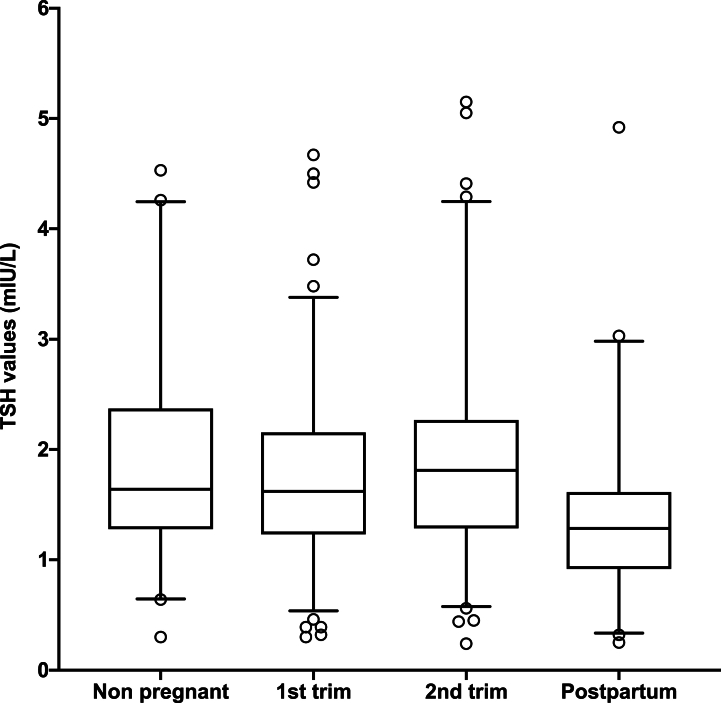
Histogram: Variation of TSH levels according to pregnancy trimesters, postpartum and in non-pregnant women Non-pregnant: TSH values in non-pregnant women,1st trim: TSH values measured in the first trimester of pregnancy and 2nd trim: TSH values measured in the second trimester, Postpartum: TSH values measured in postpartum. Whiskers represent the 2.5 and 97.5 percentiles. Using Kruskal Wallis test to compare these groups p was <0.0001.

The lower reference limit (LRL), upper reference limit (URL), and median of TSH during the first and second pregnancy trimesters, post-partum and in non-pregnant women are presented in Fig. 2 and Table 1. The medians and RIs for TSH during the first, second and postpartum are 1.57 (0.43–3.2 mIU/L), 1.84 (0.56–4.41 mIU/L), and 1.38 (0.30–3.60 mIU/L). For the control group the RI is 0.64–4.24 mIU/L, with a median of 1.66 mIU/L. There is a significant difference between TSH values across the 2 pregnancy trimesters and the control group (p < 0.0001).

### 3.4 Correlations between TSH values during different pregnancy trimesters and postpartum

A significant positive correlation was found between TSH values form the first and second trimester (ρ = 0.53, p < 0.001), as well as values from the first trimester and those of the postpartum (ρ = 0.53, p = 0.002). Additionally, TSH values measured during the second trimester have also a significant positive correlation with those from the postpartum period (ρ = 0.65, p < 0.001).

### 3.5 Prevalence of high TSH levels in pregnant women according to the ATA and the Endocrine Society guidelines

Using the more recent ATA recommendations, among the 224 pregnant women included in the study, 6 (2.68 %) had TSH values exceeding the limit of 4 in at least one of the first and second trimesters of pregnancy [5]. However, when applying the Endocrine Society's recommendations [14], 32 women (14.29 %) had TSH levels that exceeded the recommended values for TSH in at least one trimester. Specifically, in the first trimester, 20 patients (8.93 %) had TSH levels exceeding 2.5 mUI/L, while in the second trimester, 12 patients (5.36 %) had TSH exceeding 3.0 mIU/L. These data are based on the 2012 Endocrine Society guidelines which are, even if outdated, still largely clinically used.

## 4 Discussion

Our study aimed to determine the RI for TSH across pregnancy trimesters in the Lebanese population using an indirect approach [17]. This approach yields findings that are not achievable with the manufacturer's established RIs, which are often based on less representative populations and different inclusion/exclusions criteria [19].

A total of 224 pregnant women and 103 nonpregnant women were selected based on well-defined criteria, resulting in 458 TSH values. The majority of these measurements were taken in the first trimester of pregnancy, highlighting the importance attributed by obstetricians to this crucial phase of pregnancy. The medians and RIs in the first and second trimesters of pregnancy, as well as postpartum, were respectively 1.57 (0.43–3.20 mIU/L), 1.84 (0.56–4.41 mIU/L), and 1.38 (0.3–3.6) mIU/L, while in the control group of non-pregnant women, the median and RI were 1.66 (0.64–4.24) mIU/L.

The median value of TSH in the first trimester in our study was close to the ones reported in China [11] and India [12]. Our LRL was higher than the one observed in several studied populations [7,8,16,[20], [21], [22]], but similar to the one obtained in India (Table 2) [12]. However, our URL in the first trimester was very close to those reported in the UAE [10], India [12] and Korea [15]. Similar trends were observed for the second trimester. Comparing our TSH RIs for each pregnancy trimester with those recommended by the Endocrine Society [14], we found that the LRLs and URLs obtained in our study exceeded the limits recommended by this society in the first two pregnancy trimesters. It is important to note that the 2012 Endocrine Society guidelines on management of thyroid dysfunction during pregnancy and post-partum were retracted in 2021 [23], due to being considered outdated. While these guidelines were retracted, their **RI for TSH** have continued to be widely used in clinical practice, because no new official RIs have been published yet. Our results are concordant with the findings from studies in the UAE [10], India [12], China [11], and Korea [15]. The differences between our population and the populations of the UAE, India, Korea and China on one hand, and the American population on the other hand, may be attributed to iodine deficiency, which is highly prevalent in several populations except the American one. The importance of iodine's role was illustrated in an Indian study published in 2020 [21]. This study, conducted during the "final transition phase in sufficiently iodized women," aimed to redefine the RIs for TSH after correcting for iodine deficiency. The TSH RIs found in this study showed URLs and LRLs lower than all previous Indian studies. This is the first study to show that iodine supplementation decreases TSH during pregnancy. Further studies are crucial to determine if the higher RI values in our population have clinical consequences. It is also important to note that overtreating pregnant women could potentially increase the risk of developing attention-deficit/hyperactivity disorder (ADHD) or psychiatric disorders in the child, such as autism spectrum disorders [24].

**Table 2:** Comparison of RIs for TSH with different populations.

References	TSH First Trimester	TSH Second Trimester
**Lebanon** Present study	1.57 (0.43–3.20) (n = 169)	1.84 (0.56–4.41) (n = 119)
**UAE** Khalil et al. [10]**,** 2018	1.22 (0.094–3.33) (n = 136)	**1.84 (0.052**–**4.56)** (n = 146)
**China** Li et al. [11], 2013	1.66 (0.14–4.87) (n = 1024)	N/A
**China** Zhang D et al. [22], 2018	(0.02–3.78) (n = 132)	(0.47–3.89) (n = 124)
**UK** Bestwick et al. [20], 2014	1.11 (0.06–5) (n = 16,334)	N/A
**Italy** Bestwick et al. [20], 2014	1.07 (0.04–3.19) (n = 5505)	N/A
**Poland** Kostecka-Matyja et al. [16], 2017	(0.01–3.18) (n = 172)	(0.05–3.44) (n = 172)
**India** Rajput et al. [12], 2016	1.63 (0.37–3.69) (n = 99)	1.79 (0.54–4.47) (n = 74)
**Korea** Moon et al. [15], 2015	1.15 (0.01–4.10) (n = 120)	1.55 (0.01–4.26) (n = 211)

Data are expressed in the form of median, IQR (2.5 and 97.5 percentiles), and the number n. IQR: interquartile reference, n: number of TSH, UAE: United Arab Emirates, N/A: not applicable.

We observed that TSH variation across the first and second pregnancy trimesters is similar to literature descriptions, although the values were higher. The variation in TSH during different pregnancy stages is influenced by hCG variation. hCG, produced by the placenta from the first week after conception, peaks around the 10th week of pregnancy, starts to decrease [1], and plateaus around week 20 [25]. hCG has a TSH-like effect, leading to an increase in TH and subsequently to a slight decrease in TSH. The higher levels of TSH and its re-increase during the second pregnancy trimester in our population could be explained, not only by a decrease in HCG, but also by the exacerbated iodine deficiency in our population during pregnancy. In fact, in Lebanon, the first national survey in 1993 revealed a slight iodine deficiency among school-age children [26]. However, political instability delayed the effective implementation of the Universal Salt Iodization (USI) program until 1995. Unfortunately, the program's effectiveness was inadequate in the 2013–2014 national survey, since in the median urinary iodine concentration (UIC) in school-age children was only 66 μg/L indicating ongoing iodine deficiency [27].

In our study, we detected a significant correlation between TSH in the first trimester and those in the second trimester and postpartum. This highlights the reproducibility of TSH in the same woman without known thyroid history and suggests that it may be possible to predict TSH values for the second and third trimesters from a single TSH measurement. Additionally, our findings indicate that RI for TSH in the second trimester are nearly identical to those observed in non-pregnant individuals. This suggests that, in clinical practice, non-pregnancy RI could potentially be used in the second trimester, simplifying the interpretation of thyroid function during pregnancy. However, while our study contributes to the growing body of evidence supporting this approach, a single study is not sufficient to change current clinical guidelines. Further large-scale research is needed to validate this observation and assess its potential impact on clinical decision-making.

The median and RI in our control group were 1.66 (0.64–4.24) mIU/L, close to the RI identified in the Salameh et al. [28] study in which the RI was determined in 198 healthy Lebanese women aged between 18 and 65 years with negative TPO-Ab and TG-Ab. In the latter study, the median and RI were 1.72 (0.57–4.91) mIU/L.

In our study, we observed that 14.29 % of our pregnant women had a TSH above the threshold recommended by the Endocrine Society [14], which is concordant with another Lebanese study [4]. This higher prevalence in Lebanon could be mainly explained by poor iodine status, different prevalence of positive antibodies or most probably different cutoffs of TSH used to establish the upper limit of TSH (recent ATA guidelines versus Endocrine society ones).

### 4.1 Study strengths

Our study has several strengths. It is the first Lebanese study to determine RIs for TSH during the first and second pregnancy trimester in women without known thyroid disorders. Additionally, TSH levels were measured for all the subjects using the same Roche assay, which eliminates inter individual variations related to the analytic method. Furthermore, our control group aligns with the study conducted by Salameh et al. [28], indicating that our RIs are representative of the non-pregnant Lebanese population. This supports the validity of the results obtained in our sample of pregnant women. Notably, few studies have established RIs for TSH specifically during the postpartum period.

## 5 Study limitations

The limitations of this study include its monocentric nature, since all participants were recruited from a single center. Additionally, there was a limited number of TSH values. The retrospective design of the study means that not all women systematically had two TSH measurements during their pregnancy. Another consequence of the retrospective design is the inherent risk of selection bias. However, this was mitigated by including all women who met the inclusion criteria during the specified time frame. Furthermore, TPO-Ab and TG-Ab were not measured in all participants. Finally, factors that could influence TSH levels, such as BMI and iodine status were not considered in the statistical analysis.

## 6 Conclusion

Our study has established RIs for TSH in a sample of pregnant Lebanese women. Our URL is higher than that observed in the US but is closer to the RIs observed in the UAE, China, Korea or India. These trimester-specific RIs, tailored to the Lebanese population, will undoubtedly help implement adequate management of pregnant women and avoid overtreatment. However, it remains to be determined whether these slightly higher RIs in our population have clinical consequences on the mother and child. Finally, investigating the causes of these differences will require additional research, particularly assessing the iodine status of pregnant Lebanese women.

## CRediT authorship contribution statement

**Dollen Eid:** Writing – original draft, Formal analysis, Data curation. **Nizar El Bcherawi:** Data curation. **Georges Abi Tayeh:** Validation, Conceptualization. **Nada El Ghorayeb:** Writing – review & editing, Software. **Marie-Hélène Gannagé-Yared:** Writing – review & editing, Validation, Supervision, Project administration, Methodology, Formal analysis, Conceptualization.

## Declaration of competing interest

We, the authors, declare that the manuscript titled "Reference Intervals for TSH in a Sample of Lebanese Pregnant Women" is original and has not been published before, nor is it under consideration for publication elsewhere. The objective of this study is to define, for the first time, the TSH reference intervals (RIs) for each trimester of pregnancy in healthy Lebanese pregnant women.

This retrospective study included 290 pregnancies selected from the records of an obstetric clinic at Hôtel-Dieu de France University Hospital, Beirut, from January 2021 to May 2023. A control group of 103 non-pregnant women was also included. The 585 collected TSH values were stratified by trimester of pregnancy and postpartum. Our findings show that the RIs are wider than those recommended by the Endocrine Society but are closer to the revised American Thyroid Association (ATA) recommendations. Additionally, we observed a significant correlation between TSH values in the first trimester and those in the second trimester and postpartum (p ≤ 0.001 for both comparisons).

We certify that this work has been approved by all co-authors, and there are no conflicts of interest to disclose. All ethical standards concerning human subjects were followed, and the required ethical approvals were obtained from the appropriate review board at Hôtel-Dieu de France University Hospital.

## Data Availability

Data will be made available on request.

## References

[1] Epstein F.H., Burrow G.N., Fisher D.A., Larsen P.R. (1994). Maternal and fetal thyroid function. N. Engl. J. Med..

[2] Stagnaro-Green A., Abalovich M., Alexander E. (2011). Guidelines of the American thyroid association for the diagnosis and management of thyroid disease during pregnancy and postpartum. Thyroid.

[3] Dong A.C., Stagnaro-Green A. (2019). Differences in diagnostic criteria mask the true prevalence of thyroid disease in pregnancy: a systematic review and meta-analysis. Thyroid.

[4] Ezzeddine D., Ezzeddine D., Hamadi C. (2017). Prevalence and correlation of hypothyroidism with pregnancy outcomes among Lebanese women. Journal of the Endocrine Society.

[5] Alexander E.K., Pearce E.N., Brent G.A. (2017). Guidelines of the American thyroid association for the diagnosis and management of thyroid disease during pregnancy and the postpartum. Thyroid.

[6] Männistö T., Surcel H.M., Ruokonen A. (2011). Early pregnancy reference intervals of thyroid hormone concentrations in a thyroid antibody-negative pregnant population. Thyroid.

[7] Shields B., Hill A., Bilous M. (2009). Cigarette smoking during pregnancy is associated with alterations in maternal and fetal thyroid function. J. Clin. Endocrinol. Metabol..

[8] Osinga J.A.J., Derakhshan A., Feldt-Rasmussen U. (2024). TSH and FT4 reference interval recommendations and prevalence of gestational thyroid dysfunction: quantification of current diagnostic approaches. J. Clin. Endocrinol. Metabol..

[9] Dhatt G.S., Jayasundaram R., Wareth L.A. (2006). Thyrotrophin and free thyroxine trimester-specific reference intervals in a mixed ethnic pregnant population in the United Arab Emirates. Clin. Chim. Acta.

[10] Khalil A.B., Salih B.T., Chinengo O., Bardies M.R.D., Turner A., Abdel Wareth L.O. (2018). Trimester specific reference ranges for serum TSH and Free T4 among United Arab Emirates pregnant women. Practical Laboratory Medicine.

[11] Li C., Shan Z., Mao J. (2014). Assessment of thyroid function during first-trimester pregnancy: what is the rational upper limit of serum TSH during the first trimester in Chinese pregnant women?. J. Clin. Endocrinol. Metabol..

[12] Rajput R., Singh B., Goel V., Verma A., Seth S., Nanda S. (2016). Trimester-specific reference interval for thyroid hormones during pregnancy at a Tertiary Care Hospital in Haryana, India. Indian J Endocr Metab..

[13] Lazarus J., Brown R.S., Daumerie C., Hubalewska-Dydejczyk A., Negro R., Vaidya B. (2014). European thyroid association guidelines for the management of subclinical hypothyroidism in pregnancy and in children. Eur. Thyroid J..

[14] De Groot L., Abalovich M., Alexander E.K. (2012). Management of thyroid dysfunction during pregnancy and postpartum: an endocrine society clinical practice guideline. J. Clin. Endocrinol. Metabol..

[15] Moon H.W., Chung H.J., Park C.M., Hur M., Yun Y.M. (2015). Establishment of trimester-specific reference intervals for thyroid hormones in Korean pregnant women. Ann Lab Med.

[16] Kostecka-Matyja M., Fedorowicz A., Bar-Andziak E. (2017). Reference values for TSH and free thyroid hormones in healthy pregnant women in Poland: a prospective, multicenter study. Eur. Thyroid J..

[17] Jones G.R.D., Haeckel R., Loh T.P. (2018). Indirect methods for reference interval determination – review and recommendations. Clin. Chem. Lab. Med..

[18] Pregnancy: Gestation Trimesters & what to expect. https://my.clevelandclinic.org/health/articles/pregnancy.

[19] Wang X., Li Y., Zhai X. (2021). Reference intervals for serum thyroid-stimulating hormone based on a recent nationwide cross-sectional study and meta-analysis. Front. Endocrinol..

[20] Bestwick J.P., John R., Maina A. (2014). Thyroid stimulating hormone and free thyroxine in pregnancy: expressing concentrations as multiples of the median (MoMs). Clin. Chim. Acta.

[21] Pramanik S., Mukhopadhyay P., Bhattacharjee K. (2020). Trimester-specific reference intervals for thyroid function parameters in Indian pregnant women during final phase of transition to iodine sufficiency. Indian J Endocr Metab..

[22] Zhang D., Cai K., Wang G. (2019). Trimester-specific reference ranges for thyroid hormones in pregnant women. Medicine.

[23] Corrigendum to (2021). Management of thyroid dysfunction during pregnancy and postpartum: an endocrine society clinical practice guideline. J. Clin. Endocrinol. Metabol..

[24] Korevaar T.I.M. (2020). Levothyroxine overtreatment during pregnancy is associated with a higher risk of adverse child mental health outcomes. Clin. Thyroidol..

[25] Kennedy R.L., Malabu U.H., Jarrod G., Nigam P., Kannan K., Rane A. (2010). Thyroid function and pregnancy: before, during and beyond. J. Obstet. Gynaecol. (Abingdon).

[26] Doggui R., Al-Jawaldeh H., Al-Jawaldeh A. (2020). Trend of iodine status in the eastern mediterranean region and impact of the universal Salt iodization programs: a narrative review. Biol. Trace Elem. Res..

[27] Ghattas H., Francis S., El Mallah C. (2017). Lebanese children are iodine deficient and urinary sodium and fluoride excretion are weak positive predictors of urinary iodine. Eur. J. Nutr..

[28] Salameh C., Al Achkar A., El Boustany J., Sleilaty G., Gannagé-Yared M.H. (2022). Reference intervals for thyroid-stimulating hormone, free thyroxine, free triiodothyronine, and total triiodothyronine in the Lebanese adult population. Ann. Clin. Biochem..

